# Lectures on Diseases of the Eye

**Published:** 1839-07

**Authors:** 


### Art. XII.

*Lectures on Diseases of the Eye.* By John Morgan, f.l.s., Surgeon to Guy's Hospital and Lecturer on Surgery at that Institution. *Illustrated by coloured Plates.—London,* 1839. 8vo, pp. 221.

It is some four or five years since these lectures were first announced for publication. They only appear now, the author tells us in his preface, at the request of his pupils, and not from any wish on his part to appear before the profession and the public as an author of what will, he fears, be considered by them as a very imperfect work on the subject of ophthalmic surgery generally. This admission, he trusts, will shield him from hypercriticism.

"Before I proceed to describe to you," says Mr. Morgan, "the symptoms and treatment of the diseases of the eye, I wish you to understand the particular object which I have in view, in delivering these lectures. In the first place, you will recollect that it is not my intention to describe, with the absurd minuteness of a professed oculist, each trifling deviation from the ordinary diseases of this organ, which have been unnecessarily made the subject of a separate description and of a separate name. It is my wish to generalize the subject, to show you, by pointing out the analogy of the diseases of the eye with those of other parts, that ophthalmic surgery and general surgery are one and the same science." (p. 9.)

The proposition that a well-educated surgeon is competent to treat diseases of the eye is often asserted and maintained; but it is obvious that its correctness or incorrectness must depend entirely on the meaning attached to the expression "well-educated surgeon." Undoubtedly we ought to apply the epithet to him only who is as well acquainted with diseases of the eye as of other parts of the body. Do we not meet, however, with many who are generally reputed "well-educated surgeons," whose knowledge of ophthalmic surgery is nevertheless very imperfect? "It is a melancholy truth," says our author, "that amongst the general body of medical practitioners in this country, ophthalmic surgery is too frequently neglected, notwithstanding its universally acknowledged importance." (p. 2.)

We do not suppose that it is because the general pathology of the eye has been (as Mr. Morgan appears to think, from the pains he takes to show "that ophthalmic surgery and general surgery are one and the same science,") considered different from that of the rest of the body, that the diffusion of a knowledge of ophthalmic surgery has not more universally obtained; but we believe the real cause is that the special pathology and the details of the *art* have been neither taught nor studied so generally as they ought to be. The surgeon must view the eye not only as a component part of the body, and subject to all its general diseases; but he must view it under another and an altogether peculiar light; he must view the eye in fact as a living dioptric instrument, the vital constitution of which exerts more or less influence over its purely physical constitution, and contrariwise. Hence, though the eye may be perfect as an optical instrument, yet, on account of some disease of the retina, optic nerve, or brain, the function of vision shall not be performed. On the other hand, as one condition necessary for the perfection of the instrument is the complete transparency of the lenses of which the eye is composed, and the integrity of the pupil, so opacity of one or other lens, or closure of the pupil, by preventing the rays of light from impinging on the retina, though perfectly sound, will oppose an effectual obstacle to vision. All this shows that the diagnosis and treatment of diseases of the eye demand, in some degree, a specific and peculiar consideration. It is not enough for Mr. Morgan to tell us, for instance, that every disease to which the conjunctiva is subjected, is nothing more nor less than the common disease of a mucous membrane. It must also be strongly inculcated that affections of the conjunctiva involve results different from what is found in connexion with affections of other mucous membranes.

There are peculiarities of disease requiring corresponding peculiarities of treatment in every organ more or less, and the more complicated and artificial the structure of the organ the more numerous will those peculiarities be. There may be no difference in the abstract between inflammation of the conjunctiva and of other mucous membranes, but many and important differences in the detail. More than one half of the practice in ophthalmic medicine and surgery is a practice of detail, and this depends on the nature of the organ. These details are to be learned by experience only, and in fact must constantly be attended to. It is by attention to details that the mere oculist has so often made head against the regular surgeon. Teach the student practically the details of eye-surgery as you teach him the details of compounding—the details of anatomy—the details of other branches of surgery, and you will qualify him to treat the diseases of the eye as well as any other.

We are no advocates for unnecessary distinctions and names; but we think that when a specific character can be established in any morbid state, and that character influences, directly or indirectly, the treatment, that morbid state should be distinguished not only by a separate description, but also by a name, for the sake of fixing attention on it and avoiding circumlocution. We do not mean to say that no unnecessary descriptions and names have been given by writers on ophthalmic surgery, but we think that Mr. M. underrates, and in a too sweeping way, the distinctions which have been made in the morbid states of the eye. "That which in ophthalmology," it has been remarked, and we think justly, "has been decried as unnecessary minuteness, we unfortunately fail to find in the diagnosis of the diseases of other organs."

We maintain that it is a dangerous fallacy to assert in an unqualified manner that the diseases of the eye do not differ from those of other organs. The truth is, the eye, like other organs, has its general pathology and its special pathology; and the attempt to instil into the minds of students that there is nothing special, is a true way to favour indolence. "Lax and general notions," says Mr. Pearson, in the admirable preface to his work on Surgery, "floating in the understanding, will be of little advantage until they are reduced to something limited and specific; and except knowledge be in the detail, the application of it in particular instances will be attended with almost insurmountable difficulties. He, therefore, who desires to practise surgery with probity and success, must study it both as a science and as an art; for a man destitute of principles is little better than a surgical automaton, while the man of mere erudition can only be considered as a learned spectator."

Neither our limits nor the amount of original matter in this volume authorize any very detailed notice of it. We hope, however, to return to the subject at another time. But as it is dedicated by a teacher to his pupils, it may perhaps be expected that we should state what we think of the work as a manual for the use of students. It is always an invidious task to pronounce a summary opinion of a work, because there are few which have not some excellencies as well as defects. By noticing the principal examples of each, we act as counsel for and against, leaving the judgment to the reader; but in the case of a work for students, we are in a manner compelled to be not only counsel for and against, but also judge.

Mr. Morgan successively discusses the diseases of the conjunctiva, of the cornea, of the iris, of the sclerotic, and of the retina, cataract, the malignant diseases of the eye, then the different operations for cataract and artificial pupil, and last of all entropeon, ectropeon, and the morbid affections of the lacrymal conduits. "In these, my lectures on *Diseases* of the Eye," says he, "it is not my intention to describe the effects of mechanical violence upon the organ of vision and its appendages, as these, when ophthalmic and general surgery are recognized as one science, must be considered as a subject for the lectures on wounds, contusions, &c. generally, and to enter into a description of the nature of the tumours formed in the lids, and the mode of operating upon them would be needless, as similar tumours are met with in analogous structures in all parts of the body, and described to you in the course of surgical lectures, of which these form a part." (p. 216.)

After what has been said in the foregoing part of this article, we shall not stop to enquire whether these be sufficiently valid reasons for passing over in silence, in a student's manual on the Diseases of the Eye, the injuries of the eye and tumours of the eyelids; but having stated the case, we proceed to pick out one or two specimens, for and against, preparatory to summing up.

The following we think is happily expressed and practically just:

"In active diseases of the eye, there is one general rule, which you cannot remember too well, it is never to rest contented with merely checking the progress of disease,—never if you think that you can prevent it, by pushing your remedies still further,—never allow a disease of the eye to remain stationary. In other organs, where you have neither the transparency or integrity of a delicate tissue to preserve, nor an expanded sheet of nervous matter to protect from the slightest causes of disease, this may not be a point of any very material consequence. But, in active diseases of the eye, the delay of remedies for twenty-four hours will, in some cases, prove a sufficient cause for complete disorganization of the organ." (p. 8.)

The remarks at pp. 28, 29, on a point in the anatomical history of inflammation being but a speculation, and one by no means warranted by the facts adduced (for these we think are ill observed), might have been dispensed with.

The directions laid down at p. 62, for the treatment of acute purulent ophthalmia, we believe to be the best calculated to remove present danger and prevent subsequent bad results; but the following account of granular conjunctiva, we cannot do otherwise than consider a specimen of crude pathology, based on an imperfect knowledge of the anatomy of the part. Granular conjunctiva, says Mr. Morgan, "is occasioned by chronic and irregular thickening of the membrane, accompanied more or less by the organization of adhesive matter, which previous inflammatory action had poured into the cellular membrane beneath." (p. 103.) There is no account taken here of the papillary body of the palpebral conjunctiva, nor of the circumstance that between the latter and the tarsal cartilages there is no great show of cellular membrane.

At page 76 of the present Number of this Review, there is a note commenting on the vagueness with which measurements in lines are given in English works; of this we have an example in the work before us. Thus, if we were to follow Mr. Morgan's directions, as given in the text, in making the section of the cornea in extraction, we should make the opening too small. Figures 2 and 4 of plate xv. do not tally with the text, and are, on that account, more correct guides. On the other hand, in figures 7 and 8 of plate xv. we have the cataract-needle, and in figure 6 of plate xvii. the iris-knife, represented introduced too close to the cornea, whilst the directions in the text are somewhat more correct.

In regard to the plates, eighteen in number, and containing 74 figures in all, they are in general very illustrative of what they represent; and if the artist had taken a little more pains to accomplish accuracy of drawing and to preserve proportion, the figures would have had a claim to be considered more than mere diagrams, the title by which Mr. Morgan characterizes them.

To sum up: Mr. Morgan's book is of a size large enough to have given a full exposition of the subject of which it treats, had it been drawn up in the concise, aphoristical, and even dogmatical form, which is best for a student's manual; and which, considering the perfection of our knowledge of the eye, the subject admits of. But, unfortunately, although the descriptions and remarks do not differ in the main from what is usually met with in books on the eye, they are often diffuse and indefinite, and sometimes so obscurely expressed, that, in more than one instance, our efforts to comprehend the author's meaning, we confess, were unrewarded with success. And this is rendered worse by the circumstance that, without giving any synonomy, he often makes use of names in a signification different from what is met with in the books mentioned by him as classical. Mr. M. appears to be but little acquainted with the history and literature of his subject.

So much for the mode. As to the matter of the volume, the extracts we have given (and many more of the kind we could give) show that the discussions on the general pathology and therapeutics of the eye present much that is judicious and excellent; but great defects are exhibited whenever the author descends to the consideration of the details of special pathology and therapeutics.

The preceding remarks have been dictated by no spirit of hypercriticism; and we hope that what we have said, in regard to the necessity of attending to details in the study as well as in the practice of the diseases of the eye, will not be construed into any wish, on our part, to perpetuate the "unfortunate and disgraceful separation" between general and ophthalmic surgery. The only way, we believe, to hasten the time "when the distinction between a surgeon and an oculist shall cease to exist," will be to make our young medical men oculists as well as surgeons.

---

ART. XIII.

